# Factors related to the quality of life of family cancer caregivers

**DOI:** 10.3389/fpsyt.2023.1180317

**Published:** 2023-08-04

**Authors:** Zhenya Liu, Cancan Chen, Yanli Hu

**Affiliations:** ^1^Henan Provincial People's Hospital, Henan Provincial Key Medicine Laboratory of Nursing, Zhengzhou University People's Hospital, Zhengzhou, China; ^2^School of Nursing, Guangzhou Medical University, Guangzhou, China

**Keywords:** caregivers, oncology, quality of life, cross-sectional studies, cancer

## Abstract

**Background:**

Cancer caregivers directly affect patient health outcomes. To maintain the function and health of caregivers so that patients can receive efficient care, we must pay more attention to caregivers’ quality of life in the process of caring for patients. However, the factors influencing caregivers’ quality of life are complex.

**Aim:**

To assess caregivers’ quality of life in the process of caring for cancer patients and to explore the factors associated with it.

**Design:**

This was a descriptive correlational study. A self-report questionnaire was used to anonymously collect data from one Chinese cancer hospital. The Functional Assessment of Chronic Illness Therapy-Spiritual Well-Being (FACIT-Sp-12), General Self-efficacy Scale (GSES), Positive and Negative Affect Schedule (PANAS), Connor-Davidson Resilience Scale 10 (CD-RISC-10), 24-item Caregiver Burden Inventory (CBI) and Caregiver Evaluation Questionnaire were used to measure caregivers’ spiritual well-being, self-efficacy, affective well-being, resilience, caregiver burden and quality of life. One-way analysis of variance, the Kruskal–Wallis H test and multiple regression analysis were applied to measure the factors influencing caregivers’ situations.

**Setting and participants:**

A total of 315 caregivers of cancer patients were selected by convenience sampling. All participants were invited to complete the questionnaire through a one-on-one approach.

**Results:**

The mean score for caregiver quality of life was 204.62 ± 36.61. After controlling for demographic factors, self-efficacy (β’ = 0.265, *p* < 0.01), resilience (β’ = 0.287, *p* < 0.01) and positive affect (β’ = 0.103, *p* < 0.01) were protective factors for caregivers’ quality of life. Negative affect (β’ = −0.217, *p* < 0.01) and caregiver burden (β’ = −0.219, *p* < 0.01) were negative factors. Notably, not all of these predictors can predict all dimensions of quality of life.

**Conclusion:**

Caregivers’ quality of life needs to be further improved. The results of this study may provide clues to help identify factors influencing caregivers’ quality of life and implement targeted strategies to improve their quality of life.

## Introduction

### Background

Globally, cancer is a considerable challenge because of its high morbidity and mortality. In 2020, there were 19.3 million new cancer cases and 10 million cancer deaths worldwide (1). New cancer cases in China accounted for 23.7% of new cancer cases worldwide, and deaths accounted for 30% of the world’s total (2).

Cancer is one of the leading causes of death in the population and a major burden in China (3). Based on rough calculations, more than 10,000 people are diagnosed with cancer in China every day (4). Fortunately, due to timely detection and diagnosis, combined with better screening and treatment, the survival rate of cancer patients has increased significantly, and the number of survivors is increasing. The five-year survival rate for all cancers in China is increasing (5). Cancer survivors usually rely on family members or friends to be informal caregivers to help them cope with the problems and difficulties that arise during cancer treatment. Even if one patient needs only one family member to take care of him or her, the number of family cancer caregivers in China will increase by more than 10,000 every day and more than 3.6 million every year.

Facing cancer, family caregivers always put the patient first and spend the most time taking on a range of disease-related tasks, causing an imbalance between work and caregiving (6) and even heavy financial burdens (7). With the extension of care time, caregivers suffer from excessive fatigue and stress (8), especially in the stages of advanced cancer. Advanced cancer is a form of cancer that has spread from its original site to other parts of the body. It is usually more difficult to treat than earlier stages of cancer because it has had more time to grow and spread; therefore, family caregivers’ quality of life could be increasingly affected. The factors affecting the quality of life of cancer patient caregivers are complex. A systematic literature review (9) reported that the factors affecting the quality of life of caregivers differ across the cancer survivorship phases; however, it is generally considered that psychological functioning (e.g., depression, anxiety, and grief), social support and spirituality affect the quality of life of caregivers. Previous studies also suggest that demographic factors, caregivers’ esteem and financial burden (10), self-disclosure, psychological distress (11), psychosocial needs and cancer health literacy (12) are factors influencing the quality of life of caregivers. Li et al. (13) reported that female spousal caregivers perceived poorer health-related quality of life, lower life satisfaction and decreased marital satisfaction than male spousal caregivers. Landi et al. (14) revealed that higher offspring’s unmet needs regarding parental cancer were associated with lower quality of life and that offspring’s unmet needs mediated the relationship between parental illness unpredictability and offspring’s quality of life. Considerable quantitative and qualitative research has been conducted to investigate the quality of life of informal caregivers. However, professional and policy support remain inadequate to help informal caregivers improve their quality of life.

The extant literature has shown that cancer patient caregivers still have a low quality of life (15). Therefore, continuing to assess and investigate the quality of life of informal caregivers is essential because it may help us find new and more effective interventions. The main purpose of this study was to continue to explore the factors affecting the quality of life of family caregivers and to look for new intervention ideas. Quality of life is a multidimensional concept. Cancer caregivers’ quality of life has been measured in multiple ways. For example, caregivers’ quality of life has been measured with the Caregiver Quality of Life Index-Cancer (15), Quality of Life Scale-Family Version (16), FACT-G (17) and Medical Outcomes Study Health Survey (18). This diversity of assessment tools could help researchers understand multidimensional aspects of quality of life among cancer caregivers. Quality of life consists of a range of domains to measure an individual’s overall health. Common dimensions of health used to measure quality of life include physical, psychological, social, and spiritual components (19). Considering the influence of Chinese culture on caregivers, this study used the Caregiver Evaluation Questionnaire (20) to evaluate the quality of life of cancer caregivers. This evaluation tool consists of four dimensions, namely, social life, emotional health, the caregiver-patient relationship, and caregiver performance. Social life includes caregiver well-being and caregiver lifestyle disruption. Caregiver well-being is the degree of caregivers’ certainty of their own health and living environment. Caregiver lifestyle disruption is the severity of lifestyle disorder caused by caring for patients; emotional health is the emotional health experienced in caring for patients; caregiver-patient relationships involve positive caregiver-patient interactions and connections; and caregiver performance involves caregivers providing appropriate personal and health care to patients. These four dimensions well summarize the connotation of caregivers’ quality of life, which includes psychological, emotional, social functional and spiritual aspects.

### Objectives

Therefore, in this study, self-efficacy and resilience were used to measure the psychological function of cancer caregivers. Positive and Negative Affect measures cancer caregivers’ emotions, Caregiver Burden measures their social functioning, and Spiritual Well-Being measures spirituality. We aimed to explore the status quo of quality of life among family cancer caregivers in China and explore the influence of the above variables on it. Due to the impact of COVID-19, considering the manpower, material resources and available sample resources of the research team, the participants were recruited in a single center by a convenience sampling method.

## Methods

### Study design, setting, and participants

In this single-center, descriptive correlational study, a self-report questionnaire was distributed to a convenience sample of caregivers of cancer patients (*n* = 315) in a provincial cancer hospital in one of China’s provinces. The questionnaire was mainly distributed by 5 nurses working in the cancer hospital. These nurses were members of the research team who received professional training. First, with the permission of the nursing administrator, the 5 nurses went to the ward to recruit participants one-on-one to explain the purpose of the research, to obtain permission and to explain the procedure. After obtaining a signature on the consent from the participants, the questionnaire was officially distributed to them. The participants had two ways to fill out the questionnaire: the first was to fill out the questionnaire on-site if they could; the second was to complete the questionnaire sent to them and return it to the data collector within 2 days. If the participants were unable to complete the questionnaire independently due to low visual acuity, low literacy level, etc., the researchers used a unified guiding language to objectively state the questionnaire questions and options, which were answered by the patient and selected by the investigator. Inclusion criteria: ① The patient was pathologically diagnosed with cancer and was aged ≥18 years. All patients had advanced cancer and were undergoing radiation therapy or chemotherapy, with or without surgery; ② the main caregivers were the patients’ family members, including children, spouses, brothers and sisters; 3 the caregiver was aged ≥18 years and living with the patient; 4 the patient assumed the main care responsibility during hospitalization, and the continuous care time exceeded 72 h; and 5 the subject could communicate without barriers, provided informed consent, and voluntarily participated in the research. Exclusion criteria: ① the caregiver was a paid caregiver; ② the caregiver had serious physical illness or cognitive impairment, such as ischemic heart disease, osteoarthritis and so on.

The sample size of research on variable influencing factors is mostly based on the calculation formula of measurement data sample size (N = 4U^2^_α_S^2^/δ^2^), and the standard error (S = 40.14) and allowable error (δ range (0.25S, 0.50S)) are obtained based on a pretest (21); considering a 30% loss rate due to invalid questionnaires or noncooperation to the end of the study, etc., the range of final sample size was 83 ~ 331.

### Variables and measurements

After informed consent was obtained from the participants, the questionnaire was completed when the participants were available and interested. The paper questionnaire was administered one on one. The questionnaire consisted of questions on demographic characteristics, such as the sex, age, education, and marital status of the interviewees. It also asked the caregiver about some conditions during the care of the patient, as well as the caregiver’s self-efficacy, spirituality, resilience, positive and negative emotions, care burden, and so on. These variables were measured using the Chinese versions of the relevant research tools. Before the formal investigation, a presurvey was conducted with 30 caregivers who met the inclusion criteria to measure the reliability and validity of the research tools.

### Spiritual well-being

The Functional Assessment of Chronic Illness Therapy-Spiritual Well-Being (FACIT-Sp-12) (22) was used to measure caregivers’ spiritual well-being. The tool consists of 12 items and 2 subscales: meaning/peace (8 items) and faith (4 items). The total Cronbach’s α coefficient was 0.87, and each subscale of Cronbach’s α coefficient ranged from 0.81 to 0.88 (22). Each item is given a 5-point Likert scale (Not at all-0, A little bit-1, Somewhat-2, Quite a bit-3, Very much-4). The higher the score is, the better the caregiver’s spiritual well-being. Chinese scholars (23) have verified the reliability and validity of this scale in Chinese cancer patients. Exploratory factor analysis showed that the scale extraction factor was consistent with the source scale, and the content validity was 0.90. Cronbach’s α coefficient was 0.831, the Cronbach’s α coefficient of the subscale was 0.71 ~ 0.920, and the retest reliability of each item was 0.790 ~ 0.850 after 4 weeks. The Cronbach’s α coefficient in this study ranged from 0.798 to 0.903.

### Self-efficacy

The General Self-Efficacy Scale (GSES) was used to measure self-efficacy (24). The General Self-Efficacy Scale was created to assess a general sense of perceived self-efficacy with the aim of predicting coping with daily hassles as well as adaptation after experiencing all kinds of stressful life events. In samples from 23 nations (the scale is reported to be available in 33 languages), Cronbach’s alphas ranged from 0.75 to 0.90, with the majority in the high 0.80, and the scale is unidimensional (24). It has been widely used among the Chinese population and has good reliability and validity. Exploratory factor analysis extracted one factor with 10 items, and its Cronbach’s α coefficient was 0.87, the test–retest coefficient was 0.83, and the half reliability was 0.82. The score ranges from 1 (completely incorrect) to 4 (completely correct). The total score ranges from 10–40 points; the higher the score is, the better the self-efficacy. The Cronbach’s α coefficient in this study was 0.953.

### Affective well-being

Affective well-being refers to the individual’s affective experience, including positive and negative affective experiences. This concept was measured via the brief measures of the Positive and Negative Affect Schedule (PANAS), which was developed by Watson et al. (25). They viewed positive and negative affect as two dominant and relatively independent dimensions, and they verified the reliability and validity of this scale in undergraduates and adults. The Cronbach’s α coefficient ranged from 0.84 to 0.9, and the test–retest reliability ranged from 0.84 to 0.9. The Chinese version was revised by Qiu et al., and its reliability and validity were verified in Chinese undergraduates (26). Exploratory factor analysis showed that the scale extracted 2 factors: positive affect (9 items) and negative affect (9 items), which was consistent with the source scale. Confirmatory factor analysis shows that positive emotion and negative emotion are two independent factors. The Cronbach’s α coefficient for positive affect was 0.87, and the Cronbach’s α coefficient for negative affect was 0.84. The 18 items are measured on a 5-point Likert scale (Not at all-1, A little-2, Moderately 3, Quite a bit-4, and Extremely 5). The score range is 10–50 points. The higher the score is, the higher the positive or negative affect. The Cronbach’s α coefficient of positive affect was 0.922 and that of negative affect was 0.909 in this study.

### Resilience

Caregivers’ resilience was evaluated with the Connor-Davidson Resilience Scale 10 (CD-RISC-10). Campbell-Sills et al. (27) tested the reliability and validity of the scale through exploratory factor analysis and confirmatory factor analysis in undergraduates. The Cronbach’s α coefficient was 0.85. Ye et al. (28) used a two-parameter item response model to test the single dimension of the Chinese CD-RISC-10 and analyze the items of the graded response model (GRM) in Chinese cancer patients. The Chinese version had a stable undimensional structure, and the retest reliability coefficient, cutoff point, sensitivity and specificity for the Chinese version of the CD-RISC-10 were 0.855, 21.5, 0.735, and 0.833, respectively. Based on a 5-point Likert scale ranging from 0 (never) to 4 (always), the total score ranges from 0 to 40; the higher the score is, the higher the level of resilience. The Cronbach’s α coefficient was 0.946 in this study.

### Caregiver burden

The 24-item Caregiver Burden Inventory (CBI) was utilized to evaluate caregiver burden, which was developed by Novak and Guest (29). Their sample consisted of caregivers of confused or disoriented older people, and five interpretable factors (time-dependent burden, developmental burden, physical burden, social burden, and emotional burden) resulted from principal component analysis. Cronbach’s α coefficient ranged from 0.73 to 0.86. Yue et al. (30) verified the reliability and validity of the scale in caregivers of dementia patients. Five factors were obtained by principal component analysis, which was consistent with the source scale. Cronbach’s α coefficient was 0.92, the test–retest coefficient was 0.93, and the half reliability was 0.94. The score ranges from 0 (Strongly agree) to 4 (Strongly disagree). The total score ranges from 0–96 points. The Cronbach’s α coefficient of this study’s scale ranged from 0.904 to 0.960.

### Quality of life

As an outcome variable, caregivers’ quality of life was assessed by the Caregiver Evaluation Questionnaire, which was developed by Chinese scholar Xie (20). There are 56 items in the questionnaire, and the Cronbach’s α coefficient of the scale is 0.946. Exploratory factor analysis extracted 4 factors: social life, emotional health, caregiver-patient relationship and caregiver performance. The Cronbach’s α coefficient ranged from 0.931 to 0.952. Responses are scored from 1 to 5; the higher the score is, the better the caregivers’ quality of life. In this study, Cronbach’s α coefficient for this scale ranged from 0.954 to 0.976.

### Statistical methods

The data were analyzed using SPSS software version 22.0. Descriptive data analysis, calculation of Pearson correlations between variables and calculation of the Cronbach’s α coefficient of each measure were performed. Continuous variables are expressed in terms of the mean and standard deviation. Categorical variables are expressed in absolute values and percentages. One-way analysis of variance and the Kruskal–Wallis H test were applied to compare the impact of different demographics. Multiple regression analysis was used to measure the factors influencing quality of life, social life, emotional health, caregiver-patient relationship and caregiver performance. A two-sided *p* value of less than 0.05 was considered statistically significant.

### Research ethics approval

The participating hospitals’ research ethics committees approved this study (the ethics code was 2,019,014). Before the participants completed the questionnaire, they first read and signed the informed consent form. The questionnaire was completed anonymously, and the data analyst did not participate in the administration of the questionnaire.

## Results

### Participants

A total of 331 questionnaires were issued, sixteen questionnaires were deemed invalid because 50 percent of the questions were not answered, and 315 valid questionnaires were returned, for an effective response rate of 95.17%. Of the 315 patients who responded to 315 family caregivers we surveyed, 47 developed lung cancer, 38 gastric cancer, 45 colorectal cancer, 41 esophageal cancer, 51 breast cancer, 34 cervical cancer, 39 liver cancer, and 20 relatively rare cancers (4 skin cancer, 5 bone cancer, 3 laryngeal cancer, 7 nasopharyngeal cancer, 1 epithelioid sarcoma), and more than 85% of patients had an ECOG status score of 1 or 2 [physical condition ECOG score Zubrod-ECOG-WHO (ZPS, 5 points)].

### Basic demographics

The data of 315 caregivers were analyzed, and the details are shown in Table 1. Among the caregivers, males and females each accounted for half of the sample, and 88.3% were aged ≤60 years old. A total of 87.0% of the participants were married, and 51.7% had a junior high school education or below. According to the patient’s medical records, we found that most of the patients had an ECOG score of 1 or 2 (more than half had a score of 2), which was manifested as being able to walk freely and take care of themselves, but a loss of the ability to work (half of the day, they cannot get up and be active). There were 45 cancer patients with an ECOG score of 3, and their activity status was relatively poor. All of our patients were from the radiotherapy ward, so there were no patients with particularly severe disease whose ECOG status was evaluated as 4 or above.

**Table 1:** Participant characteristics (*N* = 315).

Category		*n*	%	Category		*n*	%
Gender	Male	163	51.7	Family *per capita* monthly income (￥)	≤1,000	107	34.0
	Female	152	48.3		>1,000, ≤3,000	122	38.7
Age (years)	≤60	278	88.3		>3,000	86	27.3
	≥61	37	11.7	Number of patient operations	None	138	43.8
Education	Junior high school and less	163	51.7		One	132	41.9
	Senior middle school or Polytechnic school	62	19.7		2 or more	45	14.3
	College degree and more	90	28.6	Economic pressure on the family caused by the patient’s disease	No difficulty	6	1.9
Marital status	Single	28	8.9		Can withstand	42	13.3
	Married	274	87.0		Some difficulty	117	37.1
	Other	13	4.1		Very difficult	150	47.6
Employment information at present	Unemployed	56	17.8	Reimbursement proportion of medical expenses	<30%	48	15.2
	Employed	259	82.2		30–50%	139	44.1
Care time	1 month	118	37.5		>50%	128	40.6
	3 months	81	25.7	Religious beliefs	Yes	37	11.7
	6 months	48	15.2		No	278	88.2
	>6 months	68	21.6				

### Different demographic factors on quality of life and its dimensions

The normality test showed that the data in this study basically obeyed a normal distribution. There were 11 demographic factors in this study (see Table 1). Analysis of variance, Kruskal–Wallis test or t test showed that the demographic factors affecting caregivers’ quality of life were education, family *per capita* monthly income and reimbursement proportion of medical expenses (*p*<0.05); the factor that affects caregivers’ social life is care time (*p*<0.05); the factors that affect caregivers’ emotional health were education, care time, and family *per capita* monthly income (*p*<0.05); and the factors that affect caregiver-patient relationships were education and number of patient operations (*p*<0.05). See Table 2 for details. Only statistically significant variables are listed in the table.

**Table 2:** Univariate analysis (*N* = 315).

Category		Score of quality of life	F(degrees of freedom)/P	LSD
Education	1. Junior high school and less	199.47 ± 36.82	3.497(2, 312)/0.031	1<3
	2. Senior middle school or Polytechnic school	208.53 ± 32.28		
	3. College degree and more	211.24 ± 37.98		
Family *per capita* monthly income (￥)	1 ≤ 1,000	196.82 ± 38.61	3.900(2, 312)/0.021	1<2
	2>1,000, ≤3,000	209.82 ± 34.07		
	3>3,000	206.93 ± 36.31		
Reimbursement proportion of medical expenses	1<30%	215.77 ± 36.11	3.017(2, 312)/0.05	1>2
	230–50%	200.81 ± 39.92		
	3>50%	204.56 ± 32.20		
Category		Score of social life	F(degrees of freedom)/P	LSD
Care time	1 ≤ 1 month	69.92 ± 14.46	3.112(3, 311)/0.027	1>3 1>4
	2>1 month, ≤3 months	66.07 ± 15.12		
	3>3 months, ≤6 months	64.06 ± 18.00		
	4>6 months	63.59 ± 15.82		
Category		Score of emotional health	F(degrees of freedom)/P	LSD
Education	1. Junior high school and less	50.83 ± 12.08	4.349(2, 312)/0.014	1<3
	2. Senior middle school or Polytechnic school	53.82 ± 11.50		
	3. College degree and more	55.24 ± 11.78		
Care time	1 ≤ 1 month	56.03 ± 10.60	5.464(3, 311)/0.001	1>2 1>3 1>4
	2 >1 month, ≤3 months	51.36 ± 12.34		
	3 >3 months, ≤6 months	51.21 ± 13.13		
	4 >6 months	49.49 ± 11.95		
Family *per capita* monthly income (￥)	1 ≤ 1,000	48.82 ± 12.33	8.788(2, 312)/0.000	1<2 1<3
	2 >1,000, ≤3,000	54.66 ± 10.92		
	3 >3,000	54.69 ± 12.04		
Category		Score of caregiver-patient relationship	H/P	LSD
Education	1. Junior high school and less	46.73 ± 8.95	9.647/0.008	1<2 1<3
	2. Senior middle school or Polytechnic school	50.16 ± 7.35		
	3. College degree and more	49.26 ± 8.91		
Number of patient operations	None	46.98 ± 9.27	7.552/0.023	1<2 3<2
	Once	49.85 ± 7.98		
	2 times and more	46.60 ± 8.56		

H: Kruskal–Wallis test was used when the variance was not equal.

Only statistically significant data are listed in the table, and nonsignificant variables have been omitted.

### Bivariate analyses

The mean scores and correlations for the variables are shown in Table 3.

**Table 3:** Descriptive statistics and correlation coefficients among variables (*N* = 315).

	Mean	SD	1	2	3	4	5	6	7
1. Spiritual well-being	32.52	8.18	1						
2. Self-efficacy	26.28	7.34	0.409^**^	1					
3. Positive affect	24.52	7.05	0.352^**^	0.416^**^	1				
4. Negative affect	21.46	7.44	−0.248^**^	−0.213^**^	−0.1	1			
5. Resilience	28.43	8.67	0.578^**^	0.425^**^	0.414^**^	−0.105	1		
6. Caregiver burden	50.21	28.86	−0.027	−0.094	−0.141^*^	0.232^**^	0.245^**^	1	
7. Quality of life	204.62	36.61	0.420^**^	0.495^**^	0.382^**^	−0.363^**^	0.410^**^	−0.237^**^	1

Ne. ^**^p<0.01，^*^p<0.05.

Quality of life had significant positive correlations with spiritual well-being (*r* = 0.420, *p*<0.01), self-efficacy (*r* = 0.495, *p*<0.01), positive affect (*r* = 0.382, *p*<0.01), and resilience (*r* = 0.410, *p*<0.01) and had significant negative correlations with negative affect (*r* = −0.363, *p*<0.01) and caregiver burden (*r* = −0.237, *p*<0.01).

### Multiple regression analysis

The total score for quality of life was taken as the dependent variable, and the statistically significant demographic data, spiritual well-being, self-efficacy, positive affect, negative affect, resilience and caregiver burden were taken as the independent variables to perform hierarchical regression analysis. The results showed that self-efficacy, negative affect, resilience, caregiver burden, and positive affect explained 40.6% of the total variation. See Table 4 for details.

**Table 4:** Hierarchical regression analysis of quality of life as the dependent variable (*N* = 315).

	Model 1			Model 2		
	β	β’	*t*/*P*	β	β’	*t*/*P*
Constant term	193.861	-	41.695/0.000	160.173	-	15.947/0.000
Education	6.082	0.144	2.576/0.010	−0.593	−0.014	−0.312/0.755
Self-efficacy	-	-	-	1.324	0.265	5.161/0.000
Negative affect	-	-	-	−1.070	−0.217	−4.695/0.000
Resilience	-	-	-	1.214	0.287	5.262/0.000
Caregiver burden	-	-	-	−0.277	−0.219	−4.475/0.000
Positive affect	-	-	-	0.533	0.103	1.998/0.047
*R^2^*	0.021			0.417		
Adjusted *R^2^*	0.018			0.406		
F(degrees of freedom)/P	6.634(1, 313)/0.010			36.712(6, 308)/0.000		

The tolerance (TOL > 0.1) and variance inflation factor (VIF < 5) show that there is no collinearity problem in the regression equation.

Then, multiple linear regression analysis was carried out taking the four dimensions of quality of life as dependent variables separately and taking the statistically significant demographic data, the dimensions of spiritual well-being, self-efficacy, positive affect, negative affect, resilience and caregiver burden separately as independent variables. The results are shown in Table 5. Only statistically significant variables are listed in the table.

**Table 5:** Multiple linear regression analysis of the 4 dimensions of quality of life as the dependent variable (*N* = 315).

Dependent variables	Prediction factor	β	β’	*t*/*P*	*R^2^*	Adjusted *R*^2^	F(degrees of freedom)/P
Social life	Constant term	50.382	-	11.003/0.000	0.354	0.342	28.186(6, 308)/0.000
	Self-efficacy	0.500	0.235	4.293/0.000			
	Negative affect	−0.393	−0.187	−3.896/0.000			
	Positive affect	0.308	0.139	2.601/0.010			
	Resilience	0.407	0.226	4.138/0.000			
	Time-dependent burden	−0.419	−0.166	−3.415/0.001			
	Care time	−1.332	−0.099	−2.111/0.036			
Emotional health	Constant term	41.365	-	11.306/0.000	0.456	0.442	32.061(8,306)/0.000
	Self-efficacy	0.333	0.203	4.001/0.000			
	Negative affect	−0.403	−0.250	−5.496/0.000			
	Positive affect	0.185	0.108	2.194/0.029			
	Resilience	0.274	0.198	3.457/0.001			
	Time-dependent burden	−0.310	−0.160	−3.525/0.000			
	Meaning and peace	0.612	0.268	4.246/0.000			
	Faith	−0.647	−0.197	−3.191/0.002			
	Care time	−1.439	−0.139	−3.226/0.001			
Caregiver-patient relationship	Constant term	45.235	-	14.811/0.000	0.158	0.147	14.530(4, 310)/0.000
	Negative affect	−0.178	−0.151	−2.710/0.007			
	Self-efficacy	0.199	0.167	2.928/0.004			
	Time-dependent burden	−0.256	−0.181	−3.386/0.001			
	Meaning and peace	0.204	0.123	2.103/0.036			
Caregiver performance	Constant term	21.735	-	9.187/0.000	0.182	0.171	17.235(4, 310)/0.000
	Resilience	0.246	0.214	3.176/0.002			
	Self-efficacy	0.245	0.181	3.084/0.002			
	Emotional burden	−0.161	−0.128	−2.381/0.018			
	Faith	0.352	0.129	2.049/0.041			

β: Unstandardized regression coefficient, β’: Standardized regression coefficient.

Self-efficacy, resilience, negative affect, time-dependent burden, positive affect, and care time were the main predictors of social life and explained 34.2% of the total variation. Meaning and peace, negative affect, self-efficacy, resilience, faith, time-dependent burden, care time, and positive affect were the main predictors of emotional health, and they explained 44.2% of the total variation. Time-dependent burden, self-efficacy, negative affect, and meaning and peace were the main predictors of caregiver-patient relationships and explained 14.7% of the total variation. Resilience, self-efficacy, emotional burden, and faith were the main predictors of caregiver performance and explained 17.1% of the total variation.

## Discussion

In this study, the overall scores of caregivers’ quality of life were not high, which was similar to a study (20) conducted among family caregivers of psychiatric patients using the same instrument, but the scores were lower than a study about caregivers of neurology patients (31). This difference may be related to the different types of diseases of the patients cared for by caregivers. Furthermore, in this study, 51.7% of the caregivers were less educated, and they may have had a limited ability to obtain social resources; 87% of the caregivers were married, and they may also need to perform other family tasks, such as caring for children in addition to caring for patients; and 82.2% continued to work, and they may have had no choice but to transform their roles, living with cancer patients not only as caregivers but also bearing the economic burden of the disease, which may be one of the challenges of caregiving and may partially explain the caregivers’ poor quality of life.

The current study contributed to the evidence supporting that a high caregiver burden and negative affect are associated with worse quality of life for the cancer patient’s caregiver and that high self-efficacy, resilience and positive affect are associated with better quality of life for the cancer patient’s caregiver. However, these variables had different influences on each dimension of caregivers’ quality of life. Interestingly, spiritual well-being was positively correlated with caregivers’ quality of life, but it was not entered into the regression equation when the total score of caregivers’ quality of life was the dependent variable. More interestingly, we found that meaning, peace, and faith were predictors of emotional health; meaning and peace were also predictors of the caregiver-patient relationship; and faith was a predictor of caregiver performance.

### Self-efficacy

Self-efficacy refers to one’s belief in one’s ability to accomplish a specific task under different circumstances (32), and it has been described as a positive outcome (33) and significant change in self-efficacy with intervention (34). Previous studies have shown that cancer caregivers with high self-efficacy have a higher level of quality of life, lower level of burden (35) and higher levels of mental health (36). Similar results were obtained in this study. The findings of the present study also revealed that increasing self-efficacy predicted an improvement in social life, emotional health, caregiver–patient relationships and caregiver performance. Social life included caregiver well-being and caregiver lifestyle disruption. Caregiver well-being refers to the degree of certainty of one’s own health and living environment. Caregiver lifestyle disruption refers to the severity of lifestyle disorders caused by caring for patients; emotional health refers to emotional health in caring for patients; the caregiver-patient relationship refers to positive caregiver-patient interactions and connections; and caregiver performance refers to caregivers providing appropriate personal and health care to patients (37). Caregivers need to assist with patients’ medical issues and manage patients’ symptom health, playing multiple roles in taking care of patients (38). Their own social lives are disrupted (39), and it is difficult for them to maintain their emotional well-being (38). While caring for patients, caregivers report anxiety and depression (40). Caregivers lack positive interaction with patients because without training, they do not know how to give appropriate caring to patients because they may lack knowledge and care skills. Therefore, future studies should focus on designing self-efficacy-based interventions to improve caregivers’ quality of life.

### Resilience

According to the results of the present study, resilience is positively predictive of caregivers’ social life, emotional health and caregiver performance. This finding can be applied in clinical practice. Resilience is a process of good adaptation of an individual to adversity, trauma, tragedy, threat, or other major stress, namely, the ability to rebound from difficult experiences (41). Individuals with high resilience are characterized by optimism and high levels of self-efficacy (42). These factors may play a positive role in the process of caring for cancer patients, enabling caregivers to have positive situations. Studies have shown that the level of resilience can change through changes in family, the environment, physical and mental development, and time (43); thus, resilience could intervene to give caregivers a better quality of life. The results of this study suggest that improving caregivers’ resilience can improve their social life, emotional health and caregiver performance. Effective ways to improve caregivers’ resilience include supporting caregivers with the resources of knowledge and information, promoting caregiver well-being through positive psychological activities, allowing caregivers to vent feelings and helping caregivers acquire coping strategies through social support (44).

### Affective well-being

Affective well-being refers to an individual’s emotional response to life events, including positive affect and negative affect (45). This study found that high positive affect indicated high quality of life, but high negative affect indicated lower quality of life of caregivers. A study of 240 family caregivers who cared for adolescents with depression by Zhang et al. (46) found that positive affect could predict better physical quality of life and that negative affect could predict lower mental quality of life. The present study found similar results. This result highlights the importance of affective well-being and the role that affective well-being plays in improving the quality of life of caregivers. Positive and negative psychological feelings were generated in the process of caring for patients, and they had various psychological support needs (47). Positive emotions were conducive to improving the flexibility of emotional participants’ thinking and enhancing their frustration tolerance. Mindfulness interventions could be useful for increasing the positive feelings of caregivers (48).

### Caregiver burden

This study also revealed that caregiver burden was a negative predictor of quality of life. Further analysis found that social life, emotional health and caregiver–patient relationships were greatly affected by time-dependent burden. The impact of emotional burden on caregiver performance was obvious. These findings indicated that it would be beneficial for caregivers to take interventions that can improve quality of life. Time-dependent burden is one of the main caregiver burdens (49). By considering the clinical characteristics of both patients and caregivers, timely meeting caregivers’ care needs to reduce time-dependent burden (49) can effectively improve caregivers’ social life, emotional health and caregiver-patient relationships. To provide appropriate personal and health care to patients, reducing emotional burden is an effective strategy. Caregivers have negative emotions while caring for patients, and they desire psychological support (47). Because the caregiver burden is complex by nature, recommended interventions that are likely to reduce caregiver burden are also multifaceted, such as cognitive–behavioral therapy (CBP), acceptance and commitment therapy (ACT) and interpersonal psychotherapy (IPT) (50).

### Spiritual well-being

We found that meaning and peace were positive predictors of emotional health and caregiver–patient relationships and that faith was a negative predictor of emotional health and a positive predictor of caregiver performance. This is an interesting result, and it varied from the findings by Spatuzzi R (18), who found high levels of caregivers’ spiritual well-being, more vitality, more social activities, and high levels of mental health. This may be because the tool used to assess the spiritual well-being of caregivers was the Spirituality Index of Well-Being scale (51), which includes 2 dimensions of self-efficacy and life scheme. Meanwhile, we used FACIT-Sp-12, which includes meaning and peace and faith. Meaning and peace refer to the sense of meaning and purpose that spirituality provides, as well as a feeling of harmony and peace deriving from a connection to something larger than the self; faith may measure a dimension of spirituality that overlaps with, or is enhanced by, religion (22). The participants of this study were Chinese, and cultural differences bring about different perceptions. The Medical Outcomes Study Short Form was used to measure quality of life (52). The Caregiver Evaluation Questionnaire was used in this study. This tool mainly focuses on understanding caregivers’ social life, emotional health, caregiver-patient relationships and caregiver performance. The findings of our study also demonstrated that spiritual well-being can be drawn upon to facilitate caregivers’ quality of life. Interventions should be offered based on caregivers’ meaning and peace and faith and should focus on fostering caregivers’ self-reflection of personal value, assisting them through constant self-encouragement and summary of the experience.

### Limitations

This paper had some limitations. First, we recruited 315 cancer caregivers from one Chinese cancer hospital from June to October 2021. During 2021, our country adopted a zero COVID policy, the epidemic was well controlled, and few people were infected with the new coronavirus, whether patients or caregivers. During this period, although people’s travel was affected by epidemic control to a certain extent, the treatment and life of patients were hardly affected by the epidemic. However, each caregiver’s own feelings about the impact of the pandemic on them need further verification. It had a cross-sectional design, the directions of causal relationships between the variables were based on theoretical explanations, and longitudinal studies are necessary to verify causal effects among variables. The small sample size and convenience sampling method were other limitations. Future studies should attempt to replicate our findings with larger and more representative samples. Also, since the data were collected in self-report form, the results of the study and its external validity, were threatened by recall bias. Moreover, it is possible that the findings were influenced by culture, and the replication of this study in other cultural contexts is necessary.

## Conclusion

Caregivers’ quality of life is still poor and is influenced by psychological, emotional, social and spiritual factors. Our study suggests several important factors related to the quality of life of cancer caregivers. The findings provide a rationale for developing quality-of-life interventions.

Caregivers’ quality of life may be reduced by higher caregiving burdens and negative emotions but can also be improved by high levels of self-efficacy, mental resilience, and spiritual health. These are important findings of this study that provide clinical implications. Interventions should focus on increasing self-efficacy, positive affect, resilience, and spiritual well-being and reducing negative affect and caregiver burden, which will directly or indirectly benefit cancer caregivers’ quality of life.

## Data availability statement

The raw data supporting the conclusions of this article will be made available by the authors, without undue reservation.

## Ethics statement

The studies involving human participants were reviewed and approved by Ethics Committee of Henan Cancer Hospital. The patients/participants provided their written informed consent to participate in this study.

## Author contributions

ZL was involved in the data acquisition, analysis and interpretation of the data, and the drafting of the manuscript. CC was participated in the analysis and interpretation of the data. YH made substantive intellectual contributions to the conception of the work and the interpretation of the data and revised the manuscript. All authors contributed to the article and approved the submitted version.

## Funding

This study was funded by the Guangdong Provincial Health Commission (Grant Number A2022055).

## Conflict of interest

The authors declare that the research was conducted in the absence of any commercial or financial relationships that could be construed as a potential conflict of interest.

## Publisher’s note

All claims expressed in this article are solely those of the authors and do not necessarily represent those of their affiliated organizations, or those of the publisher, the editors and the reviewers. Any product that may be evaluated in this article, or claim that may be made by its manufacturer, is not guaranteed or endorsed by the publisher.
